# Editorial: Immigration and Mental Health in Modern Societies

**DOI:** 10.3389/fpsyt.2020.600763

**Published:** 2020-10-22

**Authors:** Joshua Breslau, Guilherme Borges

**Affiliations:** ^1^RAND Corporation, Santa Monica, CA, United States; ^2^National Institute of Psychiatry Ramon de la Fuente Muñiz (INPRFM), Mexico City, Mexico

**Keywords:** migration, mental health, refugees, parenting, family separation

The papers in this collection break new ground in the study of migration and mental health through a focus on specific populations of interest in transnational context. The studies examine new migrant populations, e.g., refugees from the Syrian civil war, and new trends within well-established migrant flows, e.g., return migration in Mexico. Two of the papers examine mental health intervention strategies. Lakkis et al. present results of a preliminary study of a parenting skills training intervention conducted under what must be extremely challenging circumstances for service delivery, not to mention research, in refugee camps located in Lebanon and Jordan for families recently dislocated from Syria. The promising findings, the careful description of the program, and the thoughtful discussion of the findings will benefit future program development and research. Falas-Bague et al. also study an intervention provided to a migrant group in two different national settings. The targets of this intervention are migrants from Latin America, and the intervention was provided in the U.S. and in Spain. The intervention is tailored to the needs of a specific group and implemented within standard health care settings. The positive evidence regarding feasibility and uptake suggest that this strategy can address some causes of disparities in treatment use. Evidence that people who had previously avoided care due to distrust of the health care system responded positively to this intervention is particularly compelling. In both papers, clinical knowledge and expertise follows the population, not the provider. Tailored clinical services do not need to be reinvented from scratch in each country to which a group moves.

The other two studies highlight the complexities of populations movements within and between countries that have yet to receive appropriate attention in the literature. Donato et al. examine mental health in relationship to cross-national and intra-national migration within a single study; they use data from a national sample in Mexico to compare those moving from rural to urban areas and returning from periods of residence in the United States. Ojeda et al. is a mixed-methods study of a group that normally only surfaces anonymously in policy discussions, individuals who migrated from Mexico to the U.S. and were subsequently deported back to Mexico by the U.S. government. The study gives voice to the perspective of deportees living in the border region, separated from their families that remain in the U.S. Together these studies demonstrate the vibrant new paths of studies of migration and mental health, increasing the breadth of the field by including new populations moving within and across national borders and increasing in depth through sustained attention to uniquely vulnerable subgroups and the consequences of migration policies.

## Author Contributions

JB and GB drafted and edited the manuscript.

## Conflict of Interest

The authors declare that the research was conducted in the absence of any commercial or financial relationships that could be construed as a potential conflict of interest.

